# Correspondence

**Published:** 1897-06

**Authors:** 


### CORRESPONDENCE.

### OUR PROFESSION.

Editor Journal Comparative Medicine, etc.:

In the December number of your excellent Journal I had the pleasure of reading an article from the pen of F. W. Hopkins, D.V.S., of Kansas City, Mo. It was entitled "Our Profession," and I need not say was ably discussed, and reflects much credit on the writer; at the same time it calls to mind the wonderful advancement which the veterinary profession has made during the last decade. It is only in its infancy, however, and the question which very naturally arises just now, and one in which all who love the profession are deeply interested, is, What will be the standing of our noble profession at the expiration of another ten years? The future, to say the least, is full of bright prospects, and doubtless will not disappoint the most sanguine expectations. The great educational privileges so liberally provided by the United States Government for the free education of the masses give our young men a great opportunity to obtain a first-class preliminary training previous to entering on a college course, and the many excellent facilities we have at hand for

"teaching the young idea how to shoot" give our young enterprising Americans, many of whom are naturally talented and energetic, opportunity to become eminent in this particular branch of science, which eventually must place us in the first rank as veterinarians—no longer second to any nation on top of the globe. We can justly feel proud of the great progress which has steadily marked our onward march, and John Bull, who has never failed to express his utter contempt for any enterprise that he is not the boasted instigator of, must begin to realize that American enterprise for the advancement of veterinary science is fast getting the upper hand of him, and that his American cousins on this side of the big pond are at least worthy of the respect due all who are properly educated in the veterinary profession. It is only a short time ago that our country was overrun with charlatans and quacks, that it is not necessary to say, were a disgrace to a prosperous and civilized nation, as well as a menace to the comparatively few educated veterinary surgeons who were in this country at that time. Many of us to-day look back and wonder why some more determined effort was not made to root them out earlier; but what was done in the early history of this country was too premature to prove a success.

If I remember right, a Boston veterinary institute was first formed by Dr. Dadd, and later on Bowler, of Cincinnati, and Jennings, of Philadelphia, organized a veterinary college; but all those well intended schemes fell through for want of the necessary support, and for no other reason than that the American people had not been educated up to the standard of realizing the absolute necessity of men being educated so as to prescribe scientifically for the lower animals as well as for the human family. But the boom came at last—*tempora mutantur, et nos mutamur in illis.* The necessity for this change was apparent. Everything was ripe for it, and that which gave impetus to the grand boom was the organization of the Illinois State Veterinary Medical Association, which occurred May 22, 1883. Previous to that happy event a gentleman of our city, by the name of Daniels, commenced the publication of a monthly journal in the interest of veterinary science, which was known as the *Chicago Veterinary Journal.* It had a wide circulation, and soon became very popular among all lovers of the horse. For some reason, however, its career was brief, but it lasted long enough to arouse the apparent apathy of the veterinary profession to a sense of duty; at the same time it stimulated the young horsemen of our nation to become veterinary surgeons, and it is only right to give Mr. Daniels due credit for the active part he took in the grand movement which followed. A number of the other States soon followed the good example set by the State of Illinois, and to-day there are but a few States in the Union that have not a well-organized veterinary association.

Soon after this general waking veterinary colleges began to spring up, and like the wild flowers that decorate our vast prairies in the spring-time and impregnate the atmosphere with their inspiring fragrance, they have increased in number very rapidly, so much so that it will be remarkable, indeed, if some of them, like the flowers of spring, do not wither, or, rather, drop out of existence; but there will be enough left to continue the good work which has been so ably commenced. We have no less than seventeen well organized veterinary colleges in the United States to-day, all fully equipped with the modern improvements of the age and the necessary equipments for instruction of all who desire to become qualified members of the veterinary profession. All of these colleges are turning out more or less new men every year to swell the ranks of the large army of veterinary surgeons which, according to Dr. Hopkins's estimate, already numbers in the aggregate something in the neighborhood of five or six thousand full-fledged veterinary surgeons; and, perhaps, it will not be considered out of place here to ask the question, Is there any danger of this enterprise, like some other enterprises of our country, suffering from overproduction? It is a question that I must leave for some one else to answer, and in the meantime suggest that it might be well to petition Congress to enact a law to prohibit any man holding the title of M.R.C.V.S. from landing on American soil; or, perhaps some who are more deeply interested may consider it prudent to commence negotiations with some of our distant neighbors on the other side of the Atlantic Ocean with a view of forming a contract to furnish them with some of our young American veterinarians who, no doubt, will do justice to the cause, wherever they go. But this will answer for a joke on some of our worthy adversaries across the big sea, who must feel jealous of our rapid growth; but we can assure them that there is no danger of any of our young army invading European territory. We have plenty of work for our experts to do at home. The field we have to work in on this great American Continent offers such inducements to the student of pathology that it is absolutely inexhaustible, and our veterinarians, many of whom have already rendered valuable services by coöperating in the renowned field of literary adventure and scientific research for the advancement of veterinary science, will find ample room to practise their chosen profession in the future as in the past. It is only the commencement of a new era, as we might say, in the history of American veterinary science, which signifies much for the near future, and the veterinary profession, especially the active members who have worked so hard to stamp out quackery and raise the profession up to the present standard, have good reason to rejoice over the grand triumph which has crowned their labor, and the people should feel thankful for the protection it affords the live-stock industries of our country.

P. S. I should have been glad to have continued this article by making some brief comments on Dr. Hopkins's amusing remarks in regard to the difficulty so often experienced by us all in forming a correct diagnosis of the boss—I presume he means the "stable boss"—whom we must all admit it a critter of very peculiar instincts, somewhat inclined to be mulish and mule-like—stubborn—self-opinionated, if you please; but as I fear that I have already occupied too much of your very valuable space, I must defer it to some more opportune time, and with your consent will later on have something to say on the subject.

JOS. D. TUTHILL, M.D., V.S.

CHICAGO, ILLINOIS.

---

EDITOR JOURNAL OF COMPARATIVE MEDICINE, ETC.

DEAR SIR: I wish to ask the question whether any one has seen a bad effect produced on a consumer of milk taken from cows just tested with tuberculin? My reason for such a question is a recent experience.

April 12th and 13th I tested Mr. Lippincott's herd of cows, registered Guernseys, in fine health (a herd of tuberculous Jerseys that were housed in the same barn had been killed a year before). After taking the temperature the next morning and finding no reaction, I declared them free from tuberculosis, and thought nothing about the milk. The manager of the farm had an infant about two months old which had been nourished by the milk; a farm-laborer was also similarly situated. After feeding the milk to the babes that day both were taken sick at night, with great pain and profuse diarrhœa, a very green discharge from the bowels; the family physician was sent for, and told both families that it was the tuberculin that produced the trouble, also that I should have cautioned the families not to have used the milk. Now, I have used tuberculin in many cases, and know the milk was used, and I have never seen such results as in this case; and yet I think there might have been something else that might have caused it. I wrote to the family physician, asking for a report of the cases and his opinion direct, but as yet he has not answered it. The infants had been healthy before, and have not been fed milk since, and have no more trouble.

Very truly yours, W. H. RIDGE.

TREVOSE, May 10, 1897.

P. S. Since the above was written a letter has been received which I deem best to publish in full.

MY DEAR DOCTOR: Yours at hand, and contents noted. I did not intend to *criticise* you in the least; indeed, did not know it was you that had fixed the cows, and seek to apologize for same, as it was purely unintentional. But the fact remains, *it* or something else—and the something else seems to be wanting, as the children had no other food but the cow's milk—produced indigestion, followed by vomiting and diarrhœa, which were persistent as long as we used the milk, and subsided instantly when the food was changed. Now, I do not say positively it was the milk, but it seemed a strange coincidence: two cases at the same time and under the same circumstances. What do you think? Very truly yours,

J. SIBBALD.

FOX CHASE, May 11, 1897.

---

[*Exact copy of letter received by an agent, Arkansas and Choctaw Railway.*]

GOODLAND, ARK., July 31, 1896.

MR. J. W. AMERMAN,

Mgr. Arkansas & Choctaw Ry. Co., Texarkana, Texas.

DEER SUR: Boute the 4th the trane struck mye bool which was in mi pastyre at 39 mile post; he is kut so bad I think he will surly di. i have dun ivry thing i cood to save him, don't think I ken hardly. Wun of his hawns is tuk of, and he is kut in his belly a site to see, hit tuk of a peas uf hyd bout wun feet squair between his nebul and pekir hoal and has tuk out boath his seads and most of his bags levin not enuf to be of eny use hardly. i fere it is no chants fur him to git well. He curtainly is onqualified to atkt as a bool eny more. i tell you he is no better than ded and i wish you wood make your section boos repot him as ded an pay me fur him as an animel kild on the rale rode. he is curtainly onqualifiede for a bool an is tu noked up for a stere. and is tu tuf an ranke fur befe. Please sur hav him repoted ded an pay me fur him as a valuable beest. Yors truly,

JOHN JACKSON.

P. S. Donte fale to repote him sur and repote him a ded bool in good survic at boolin time.

P. S. 2. i sent this in haste by passenger neebor Sam Jones to the city. Rite me to Goodland sune as possibul.

